# Enhancing synchronization stability in a multi-area power grid

**DOI:** 10.1038/srep26596

**Published:** 2016-05-26

**Authors:** Bing Wang, Hideyuki Suzuki, Kazuyuki Aihara

**Affiliations:** 1 School of Computer Engineering and Science, Shanghai University, No. 99 Shangda Road, Baoshan District, Shanghai 200444, P. R. China; 2 Institute of Industrial Science, The University of Tokyo, 4-6-1 Komaba, Meguro-ku, Tokyo 153-8505, Japan; 3 Graduate School of Information Science and Technology, The University of Tokyo, 7-3-1 Hongo, Bunkyo-ku, Tokyo 113-8656, Japan

## Abstract

Maintaining a synchronous state of generators is of central importance to the normal operation of power grids, in which many networks are generally interconnected. In order to understand the condition under which the stability can be optimized, it is important to relate network stability with feedback control strategies as well as network structure. Here, we present a stability analysis on a multi-area power grid by relating it with several control strategies and topological design of network structure. We clarify the minimal feedback gain in the self-feedback control, and build the optimal communication network for the local and global control strategies. Finally, we consider relationship between the interconnection pattern and the synchronization stability; by optimizing the network interlinks, the obtained network shows better synchronization stability than the original network does, in particular, at a high power demand. Our analysis shows that interlinks between spatially distant nodes will improve the synchronization stability. The results seem unfeasible to be implemented in real systems but provide a potential guide for the design of stable power systems.

Electric power grids can operate normally only if the total electricity demand matches the total supply from all the power plants in the grid. All generators of the network have to be stabilized at the same frequency even after a perturbation. A disruption in synchronization may cause the malfunction of generators and the outages of power grids with cascading catastrophic failures of power plants, as have been observed at New York in 1965 and at the Western American network in 1996^1^.

Synchronization stability is strongly affected by the distribution of power demand^2^,^3^,^4^. A decentralized grid is found to enhance the network robustness against structural damage, while it becomes more sensitive to the dynamical perturbations^2^,^3^. Usually, due to fluctuation of the real power demand, the robustness of load nodes is also used to measure the network robustness to the fluctuation^5^. On the other hand, network topology plays an important role in the stability of network synchronization. As a paradoxical example, the additionof a transmission line or the increase of line capacity may weaken the synchronization, which is known as Braess’s paradox phenomena^6^,^7^. Synchronization stability can be further improved by relating the system parameters to the network topology. Motter *et al.* derived the master stability function in terms of the eigenvalues of the coupling matrix and the network parameters^8^. By tuning the dynamical parameters such as the damping coefficients and the feedback gains, to match the network topology, the synchronization stability could be optimized.

The information and communication technologies have altered the dynamics of real power systems. In order to maintain synchronization in a power grid, the operation is based on the controlled areas. A power controlled area is a part of the system under the supervision of a control center, where operators balance supply and demand without creating overloads as well as underload. In practice, generators are often controlled by governors; the mechanical power input to generators is adjusted according to the generator’s frequency as self-feedback control^9^,^10^. It is also feasible to take the information of neighboring generators into account and adjust the power input to the generator accordingly^9^. Thus, generators can communicate with each other through a communication network. Since the communication network itself is not necessarily the same as the substrate network, building a reliable communication network where each pair of connected generators can efficiently exchange information, is necessary^11^. From the view point of complex networks, the communication network and the power grid can be represented as a multiplex network^12^. The layer of the communication network influences the dynamics of the power grid.

Power grid networks are often composed of a number of areas, which are densely connected internally and weakly interconnected with each other. This is because generators and loads are often spatially connected and the lengths of transmission lines are usually limited. The dynamical processes such as synchronization^13^,^14^,^15^,^16^ and diffusion processes^17^ on local subnetworks can further affect the dynamics on the entire system. For instance, phenomena of breathing synchronization where two groups synchronize at different frequencies can also emerge^15^.

The frequency control of generators has to take the network structure into account. In order to enhance the synchronization stability, building an efficient communication network where each pair of connected generators can exchange information is necessary. In this paper, we investigate the steady-state stability of an interconnected power grid network under different control strategies. By the steady state stability, we mean the local stability of a system, i.e., its ability to return to the pre-perturbed state after a small disturbance is introduced. This is different from the basin stability, where we consider large perturbation occurring in the network^18^,^19^,^20^,^21^. Based on the phenomena of multi-area power grid networks, we investigate the enhancement of the synchronization stability in terms of the control strategies and the topology design of network interlinks. Regarding the control strategies, we compare three possible control strategies. The first one is the self-feedback control, where the governors adjust the power input to the generator according to its frequency; second, a local feedback control is achieved by building a local communication network based on the local network topology of the power grid, where governors adjust the power input according to the information of its neighboring generators in the communication network; finally, a global control of the entire network is assumed to be built on the communication network of generators located at different subnetworks. We derive the master stability function for the swing equations with the incorporation of these control strategies and build the communication network accordingly. Although a similar idea of designing stabilizing controllers was previously studied^22^, our emphasis is to build a proper communication network by relating the oscillators’ states to the network connectivity.

The design of a real power grid is practically a consequence of the trade-off between the length of transmission lines and the degree of stability, since longer grid lines often need enormous cost. The way of adding interlinks between different areas is highly related to the network synchronizability. A pattern of high-degree nodes connecting with high-degree nodes has been found to promote synchronization most^23^. In order to relate the interconnected network to the synchronization stability, we investigate the enhancement of the network synchronization stability by changing the network interlinks. Although the optimized network and the original network are different in topology and their respective steady states are different, it is still possible to measure their ability to return to their own pre-perturbed states. By adding interlinks for the optimized routine, the optimized network shows better stability than the original network does for a range of power demand. By this study, we clarify the impacts of the network structure on the synchronization stability and get insights on the design of real power grid networks.

## Results

### The model

A typical swing equation is often used to describe the dynamics in a power grid and can be taken as a second-order Kuramoto model with inertia^24^. The swing equation that governs the mechanical dynamics of generator *i* is given by


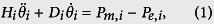


where *i* = 1, …, *n*, and *n* is the number of machines in the network; *H*_*i*_ and *D*_*i*_ are the inertia and damping coefficients of generator *i*, respectively. *P*_*m*,*i*_ is the mechanical power injected in *i* and *P*_*e*,*i*_ is the electric power output of *i*; *θ*_*i*_ is the rotor angle of generator *i* in respect to a synchronously rotating reference frame in radians. Equation (1) can be converted to a set of first-order differential equations as follows:


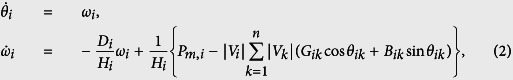


for *i* = 1, …, *n*, where *θ*_*ik*_ = *θ*_*i*_ − *θ*_*k*_ represents the phase difference between generators *i* and *k*; |*V*_*i*_| and *θ*_*i*_ are the voltage and the phase of generator *i*, respectively; *ω*_*i*_ is the phase frequency of generator *i*. The admittance matrix *Y* is composed of complex numbers, expressed as *Y*_*ik*_ = *G*_*ik*_ + *jB*_*ik*_, with *j*^2^ = −1, where *G*_*ik*_ and *B*_*ik*_ are conductance and susceptance between generators *i* and *k*, respectively.

In what follows, we assume that a power grid network is composed of two subnetworks ‘a’ and ‘b’, whose numbers of nodes are *n*_*a*_ and *n*_*b*_, respectively. We denote the set of nodes in the network as 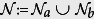, where 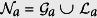, 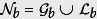, and 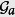 (or 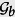) and 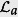 (or 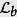) denote the set of generators and that of loads in subnetwork ‘a’ (or subnetwork ‘b’). We further denote the set of generators in the network as 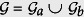. The analysis of a network with two subnetworks here can be naturally extended to the one that contains an arbitrary number of subnetworks. The dynamics of the entire system, including the load nodes, can be reduced to the dynamics of a system composed only of the generators (see Supplementary Information S1). Then, the swing equations for the entire system are given by


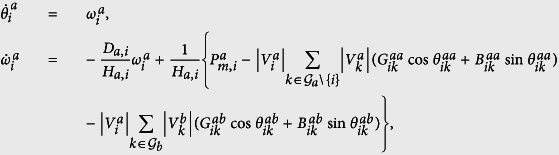



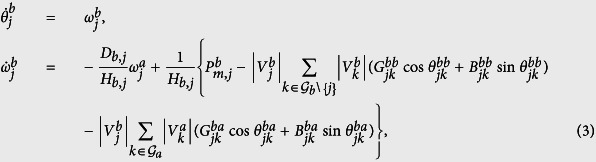


for 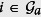 and 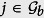. The matrix *G*^*aa*^ (or *G*^*bb*^) is the conductance matrix in subnetwork ‘a’ (or subnetwork ‘b’), while *G*^*ab*^ is the conductance matrix that connects generators in subnetwork ‘a’ with those in subnetwork ‘b’; the matrix *B*^*aa*^ (or *B*^*bb*^) is the susceptance matrix that connects the generators in subnetwork ‘a’ (or subnetwork ‘b’), and *B*^*ab*^ (or *B*^*ba*^) is the susceptance matrix that connects the generators in subnetwork ‘a’ (or subnetwork ‘b’) with the generators in subnetwork ‘b’ (or subnetwork ‘a’), see Supplementary Information S2. In the following, based on equation (3), we carry out the steady-state stability analysis with the incorporation of different control strategies.

### Steady-state stability with self-feedback control

Maintaining the rotator frequency is a prerequisite for the stable operation of power systems. Usually, a self-feedback control of rotator is often implemented by governors^9^. Thus, the mechanical power input into generator *i*, *P*_*m*,*i*_, for 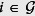, is adjusted in order to keep the frequency close to the standard frequency. Assume that the mechanical power input at generator *i* in subnetwork ‘a’ is controlled with the derivative of the phase frequency 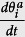, that is,


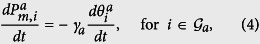


where *γ*_*a*_ > 0 is the feedback gain of generators in subnetwork ‘a’. The equation is rewritten as


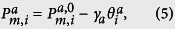


where 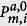 is the constant power input into generator *i*.

We denote the equilibrium solution of equation (3) as 
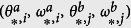
 for 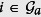 and 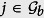, and 
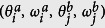
 is the state obtained by the perturbation around the equilibrium expressed as 
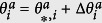
, 
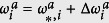
, 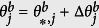, 
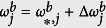
 (see Supplementary Information S3 for the details). By introducing vectors **X**_**1**_ and **X**_**2**_ defined as


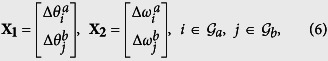


we obtain the following equations (see Supplementary Information S4 for the details):


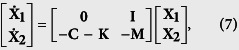


where **0** is the zero matrix and **I** is the identity matrix; the matrices **K** and **M** are the self-feedback control matrix and the damping matrix (see Supplementary Information S4). The matrix **C** is an (*n*_*a*_ + *n*_*b*_) × (*n*_*a*_ + *n*_*b*_) Laplacian matrix representing the topology of subnetwork ‘a’, subnetwork ‘b’, and the network interlinks between them, which relate to the synchronized state, defined as


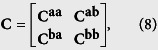


where


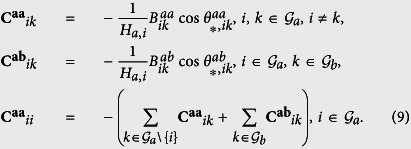


The matrix **C**^**bb**^ can be defined in a similar way as **C**^**aa**^. We also assume that the network is undirected, so we have **C**^**ba**^ = (**C**^**ab**^)^*T*^. Since **C** is the Laplacian matrix, it can be further diagonalized as **J** = **QCQ**^−**1**^, where **Q** is composed of the eigenvectors of **C**, and **J** is the diagonal matrix of the corresponding eigenvalues, 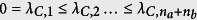. With the transformation **Z**_**1**_ = **Q**^−**1**^**X**_**1**_ and **Z**_**2**_ = **Q**^−**1**^**X**_**2**_, equation (7) is equivalent to


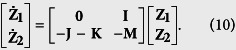


The synchronization stability is determined by the following eigenvalues (see Supplementary Information S4):


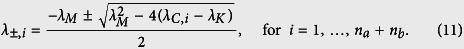


In order to keep stable synchronization, the real parts of all the eigenvalues should be less than zero, that is,


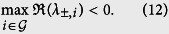


For simplicity, we denote 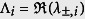 for 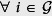 and Λ_*max*_ = max_*i*_ Λ_*i*_. The synchronous stability can be enhanced by reducing Λ_*max*_. In equation (11), the eigenvalue *λ*_*M*_ represents the effect of the inertia and the damping coefficients, which can be tuned by the parameters *H*_*a*,*i*_ (or *D*_*a*,*i*_) for 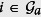 and *H*_*b*,*i*_ (or *D*_*b*,*i*_) for 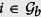. *λ*_*C*,*i*_ represents the role of network structure at the synchronized state, while *λ*_*K*_ is determined by the self-feedback gain at generators. If the network structure is fixed, the combination of the parameters *H*_*a*,*i*_ (or *H*_*b*,*i*_) and *γ*_*a*,*i*_ (or *γ*_*b*,*i*_) can cooperate to minimize Λ_*max*_.

Let us denote 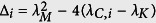, for *i* = 1, …, *n*_*a*_ + *n*_*b*_. If Δ_*i*_ < 0, the stability condition Λ_*i*_ < 0 for 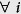, is trivial, since *λ*_*K*_ < 0 is always satisfied. The maximum value of Λ_*i*_, Λ_*max*_, is given by 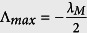 and does not change even if *λ*_*K*_ is further decreased by tuning the parameters *γ*_*a*,*i*_ and *H*_*a*,*i*_ for 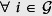.

If Δ_*i*_ > 0, Λ_*i*_ is negative and decreases with the increase of *λ*_*C*,*i*_ − *λ*_*K*_, which is determined by *λ*_*C*,2_ − *λ*_*K*_, where *λ*_*C*,2_ is the smallest nonzero positive eigenvalue of the matrix **C**. To enhance the synchronization stability, one possible way is to reduce *λ*_*K*_ by increasing the intensity, controlled by the parameter *γ*_*a*_ or *γ*_*b*_. For instance, assume that *γ*_*a*_ = *γ*_*b*_ = *γ* and *H*_*a*,*i*_ = *H*_*b*,*i*_ = *H* for 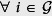. Then, the minimum value of the feedback gain *γ*_0_ can be solved with the following equation:


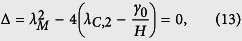


from which we obtain 
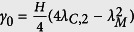
. The other way to improve synchronization stability is to increase *λ*_*C*,2_ as much as possible, which can be achieved by changing the inter-subnetwork structure. We will discuss it later.

**Figure 1:**
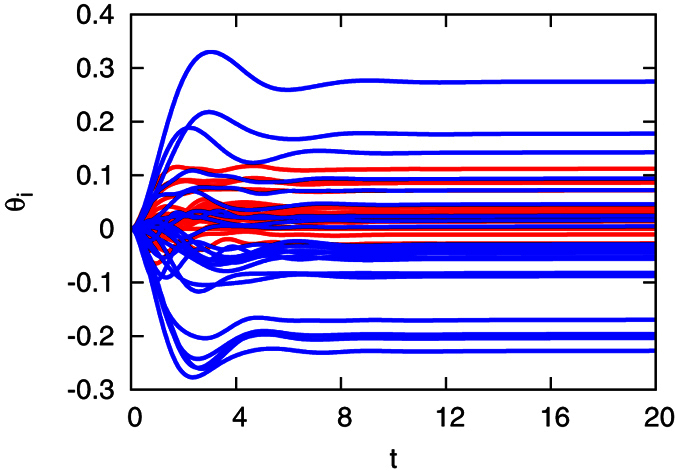
The dynamics of the generators in the Japan power grid network. Generators in the two areas are shown in blue and red, respectively. The parameters are set as *H*_*a*,*i*_ = *H*_*b*,*i*_ = 1, *D*_*a*,*i*_ = *D*_*b*,*i*_ = 1, and 
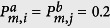
 for 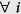, 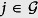. *G*_*ij*_ = 0 and *B*_*ij*_ = 10 if there is a link between node *i* and *j*; *G*_*ij*_ = 0 and *B*_*ij*_ = 0 otherwise. We employ the fourth-order Runge-Kutta method for the generators with equation (S3) and Newton’s method for the load equation (S4) alternately (see Supplementary Information S2).

We performed the numerical experiments using the data of the eastern Japan power grid network^5^, which is composed of the Tokyo area (subnetwork ‘a’) and the Tohoku area (subnetwork ‘b’), respectively. When the frequency control of generators is absent, the network converges to a phase-locked state with an appropriate value of the power demand. In Fig. 1, the phases of the generators in the two areas (the blue and red curves) are calculated with equations (S3) and (S4) in Supplementary Information S2.

**Figure 2:**
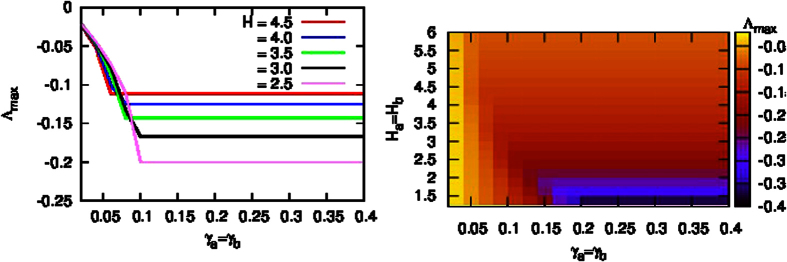
(Left)The real part of the largest eigenvalue Λ_*max*_ versus *γ* for different values of *H* with the self-feedback control strategy. The parameters are set as *γ*_*a*,*i*_ = *γ*_*b*,*i*_ = *γ* and *H*_*a*,*i*_ = *H*_*b*,*i*_ = *H* for 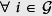. (Right) Λ_*max*_ versus *γ*_*a*,*i*_ (or *γ*_*b*,*i*_) and *H*_*a*,*i*_ (or *H*_*b*,*i*_) for 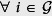.

Figure 2 shows the real part of the largest eigenvalue of the matrix **L**, Λ_*max*_, under the self-feedback control strategy. For simplicity of demonstration, we assume that the feedback control gains in the two subnetworks ‘a’ and ‘b’ are the same, i.e., *γ*_*a*,*i*_ = *γ*_*b*,*i*_ = *γ* for 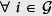. Unless specified explicitly, we assume that the inertia coefficient and the damping coefficient are constant, i.e., *H*_*a*,*i*_ = *H*_*b*,*i*_ = *H*, *D*_*a*,*i*_ = *D*_*b*,*i*_ = 1 for 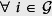. We observe that Λ_*max*_ decreases with the increase of the control gain, *γ*, and reaches the minimum value 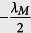 at *γ*_0_. On the other hand, Λ_*max*_ decreases with the decrease of the inertia coefficient *H*.

**Figure 3:**
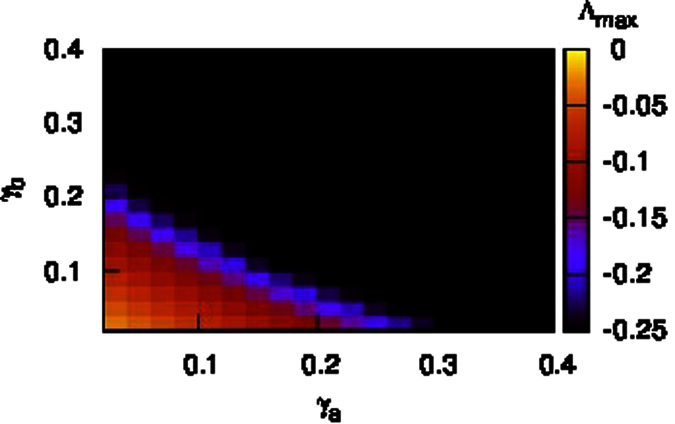
The real part of the largest eigenvalue Λ_*max*_ versus *γ*_*a*_ and *γ*_*b*_ with the self-feedback control strategy. The parameters are set as *H*_*a*_ = *H*_*b*_ = 2, *γ*_*a*,*i*_ = *γ*_*a*_ , and *γ*_*b*,*i*_ = *γ*_*b*_ for 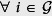.

Figure 3 shows the real part of the maximum eigenvalue Λ_*max*_ versus *γ*_*a*_ and *γ*_*b*_. We find that Λ_*max*_ decreases with the increase of the feedback gain, *γ*. If *γ*_*a*_ ≥ *γ*_*a*,0_ and *γ*_*b*_ ≥ *γ*_*b*,0_, Λ_*max*_ reaches the minimum value and keeps it. Furthermore, we find that *γ*_*b*,0_ < *γ*_*a*,0_, which indicates that the self-feedback control of generators in subnetwork ‘b’ is more efficient than that in subnetwork ‘a’ by yielding smaller control strength.

### Steady-state analysis with local- and global-feedback controls in communication networks

It is important to maintain the standard frequency at a constant value in electric power systems. If the power demand exceeds a critical value, the standard frequency cannot be maintained. In the real power grids, the frequency is often controlled by local feedback using governors and a global regulation by the control center. Based on the network structure of a multi-area power grid, we compare two kinds of control strategies. One is the local control of generators in subnetwork ‘a’ (or subnetwork ‘b’), while the other is the global control of generators located at different subnetworks.

**Figure 4:**
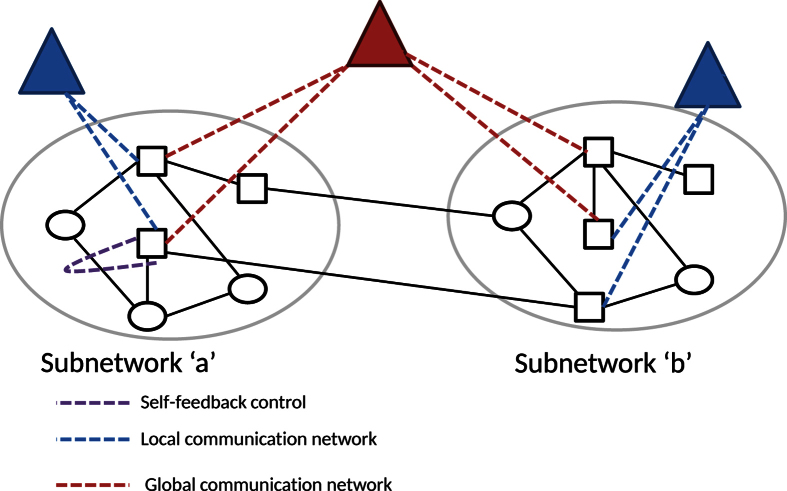
Diagram of a communication network built on a network composed of two subnetworks ‘a’ and ‘b’. Self-control of generators (the purple dashed line); a local communication network on subnetwork ‘a’ or ‘b’ (the blue dashed lines); the global communication network connecting generators in subnetwork ‘a’ with those in subnetwork ‘b’ (the red dashed lines). The control centers are denoted by triangles. Each pair of generators connected to the control center can exchange the information.

Local control of generators can be accomplished by building a communication network, where the mechanical power input to generator *i* is adjusted according to the received information from neighboring generators within the same area. For instance, in Fig. 4, the local control center is built in subnetwork ‘a’ (the blue triangle), and generators connected to the control center can exchange information and adjust the power input accordingly. For a multi-area power grid, building a reasonable communication network is fundamental to efficiently control the frequency of generators. Intuitively, a complete communication network, where each generator can receive information from all the other generators, would be most efficient. However, in actual power grids, it is hard to build such a completely connected communication network due to the extremely high cost. Hence, building an effective communication network with less cost is necessary.

The issue of building a communication network in subnetwork ‘a’ can be formulated as a problem to find a communication matrix 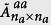, such that


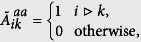


where 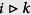 indicates that generator *i* is connected with generator *k* by the communication network of subnetwork ‘a’. The mechanical power input to generator *i* in subnetwork ‘a’ is then given by


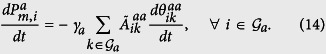


Our goal is to find a matrix 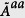 such that the synchronization stability can be improved most. In a similar way, the efficient local control of subnetwork ‘b’ is achieved by finding an efficient communication network matrix 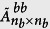. Finally, the efficient global control of generators is achieved by building a communication network matrix 
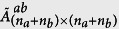
 such that generators in subnetwork ‘a’ can communicate with generators in subnetwork ‘b’, see Fig. 4 (the red dashed links). In the following, we only present the stability analysis with the local control in subnetwork ‘a’ (see Supplementary Information S5 for the details).

Equation (14) is rewritten as


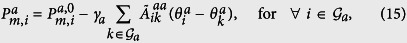


where 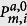 is a constant mechanical power input.

With similar analysis as we did for the self-feedback control and by setting the variables as in equation (6), we obtain an equation analogous to equation (7) for variables **X**_**1**_ and **X**_**2**_ as follows:


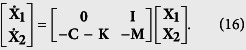


The local feedback control matrix **K** is defined as


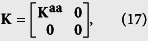


where


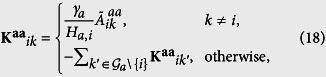


for *i*, 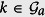, and 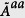 denotes the communication matrix in subnetwork ‘a’; **K**^**aa**^ is the Laplacian matrix similar to the matrix **C**.

The analysis here can be processed in the same way as that in the self-feedback control. However, the main difference between the two strategies is that in the self-feedback control strategy, **K**^**aa**^ is a diagonal matrix, while in the local control strategy, **K**^**aa**^ is a Laplacian matrix with the eigenvalues 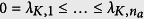. The number of zero eigenvalues depends on the number of components in the communication subnetwork ‘a’.

By diagonalizing the matrix **C** + **K**, we obtain the matrix **L** as


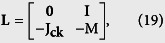


The synchronization stability is determined by the eigenvalues of **L**,


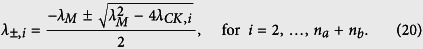


Again, we denote the real part of *λ*_±,*i*_ as Λ_*i*_, the maximum of which as Λ_*max*_, and 
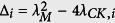
. The condition Λ_*i*_ < 0 is always satisfied because *λ*_*CK*,*i*_ > 0 is always satisfied. If Δ_*i*_ > 0, Λ_*i*_ decreases with the increase of *λ*_*CK*,*i*_. Therefore, in order to improve the synchronization stability, the communication links have to be chosen such that Λ_*max*_ is minimized or equivalently, *λ*_*CK*,2_ is maximized.

If a global control of generators is implemented, a global communication matrix that connects generators in subnetwork ‘a’ with generators in subnetwork ‘b’ is established. Then, the matrix 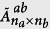 is defined as:


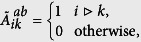


for 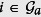, 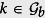. The control matrix **K** can be defined from the relation to 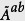 (see Supplementary Information S5).

**Figure 5:**
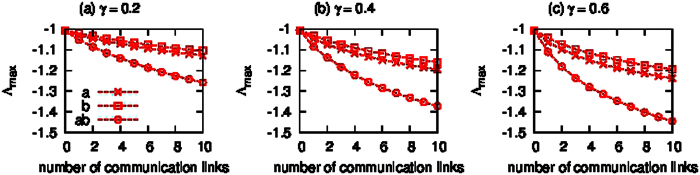
Comparison of the improved real part of the eigenvalue, Λ_*max*_, obtained by establishing the communication networks with different three feedback control strategies: namely, the local control of subnetwork ‘a’ (crosses), that of subnetwork ‘b’ (empty squares), and the global control of the network by connecting generators in subnetwork ‘a’ with generators in subnetwork ‘b’ (empty circles). All oscillators are assumed to be controlled with the same strength, i.e., *γ*_*a*,*i*_ = *γ*_*b*,*i*_ = *γ*_*ab*,*i*_ = *γ* for 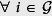; (**a**) *γ* = 0.2; (**b**) *γ* = 0.4; (**c**) *γ* = 0.6. The other parameters are set at *H*_*a*,*i*_ = *H*_*b*,*i*_ = 1 for 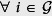. The total number of the communication links is 10.

As an example, we used the network structure of the power grid in eastern Japan^5^, and built the local communication network in subnetwork ‘a’ (Tokyo), subnetwork ‘b’ (Tohoku), and the global communication network, respectively, by following the algorithm in Supplementary Information S5. In Fig. 5, we compared the largest real part of the eigenvalues Λ_*max*_ with the local control strategy of subnetwork ‘a’ (crosses), that of subnetwork ‘b’ (squares), and the global control of the entire network (circles) for different feedback gains with *γ* = 0.2, 0.4, and 0.6, which are assumed to be the same for all the generators, i.e., *γ*_*a*,*i*_ = *γ*_*b*,*i*_ = *γ*_*ab*,*i*_ = *γ* for 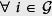. The total number of the communication links is set as 10. Figure 5 shows that Λ_*max*_ decreases as the number of communication links increases with all the control strategies that we tested. In particular, the global control of generators located at different subnetworks is most efficient, while the local control of generators in subnetwork ‘a’ is more efficient than that in subnetwork ‘b’.

**Figure 6:**
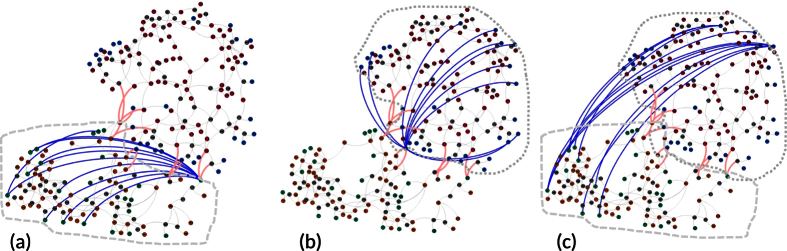
Communication networks built based on different control strategies. (**a**) Local control of generators in subnetwork ‘a’ (Tokyo, generators are denoted by green circles); (**b**) Local control of generators in subnetwork ‘b’ (Tohoku, blue circles); (**c**) Global control of generators in different subnetworks. The links in pink represent the interlinks between the two subnetworks. The links in blue are the communication links being built. The gray dashed curves show the controlled areas. The parameters are set at *γ*_*a*,*i*_ = *γ*_*b*,*i*_ = *γ*_*ab*,*i*_ = *γ* = 0.2 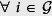. The total number of the communication links is 10.

In Fig. 6, we show the local communication network built in subnetwork ‘Tokyo’ ((a)), that in subnetwork ‘Tohoku’ ((b)), and the global communication network that connects generators in the different two subnetworks ((c)), respectively. We find that for the local-feedback control strategy (Fig. 6(a,b)), the communication networks are centralized where hub controllers are formed. This can be easily understood since the appearance of hub nodes benefits the communication among nodes, as has been observed in other dynamical processes, such as the spread of message and infectious diseases. For the global control strategy (Fig. 6(c)), we observe that most of the communication links are those whose end nodes are spatially distant.

### Enhancement of synchronization stability by changing the interlinks

It is well known that the system synchronization strongly depends on the network topology^14^,^25^,^26^. Intuitively, the more interlinks there are between subnetworks, the more synchronizable the network is. However, due to the economical considerations, the number of interlinks has to be very limited in the actual power systems. Therefore, building effective network interlinks between subnetworks is fundamental in the design of the real power systems. In order to understand the impact of network interlinks on the synchronization stability, we investigate the improvement of the synchronization stability by changing the network interlinks.

The variational equation for node *i* is rewritten as:


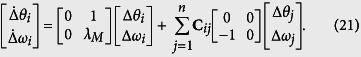


This is in the same general form with the variational equation for the coupled oscillators at the synchronous state *s*^27^ as follows:


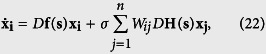


for *i* = 1, …, *n*, where 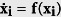 describes the node dynamics. *D***f**(**s**) and *D***H**(**s**) are both constant matrices; *σ* is the coupling strength; **C**_*ij*_ in equation (21) corresponds to *σW*_*ij*_ in equation (22). The Laplacian matrix *W* = *D* − *A*, where *D* is the diagonal matrix with the row sums of *A* as the diagonal elements, and *A* is the adjacency matrix of the network. The ascending order of the real parts of the eigenvalues is given as 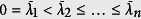. The larger 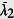 is, the more synchronously stable the network is.

We apply perturbation analysis to improve 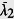 by adding interlinks appropriately in the two interconnected networks. Assume that the weight of a link connecting nodes *i* and *j* is *w*_*ij*_ > 0. When the two subnetworks ‘a’ and ‘b’ are isolated, the weighted Laplacian matrix is given by


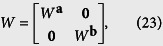


where *W*^**a**^ and *W*^**b**^ represent the weighted Laplacian matrices of the subnetworks ‘a’ and ‘b’, respectively. Since *W* is a real symmetric matrix, it has *n*_*a*_ + *n*_*b*_ real eigenvalues, which are ordered as 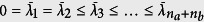, where 0 is the eigenvalue with multiplication 2 due to the two isolated subnetworks with the eigenvectors all of whose components are 1.

The second nonzero eigenvalue of *W*′ = *W* + Δ*W* is perturbed around 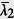, i.e., 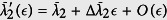, where *ϵ* is the coupling strength. By setting the eigenvector of 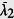 as 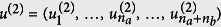, where the superscription denotes the Fiedler vector while the subscription denotes the node index. Since Δ*W* is semi-definite, we have 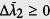. The larger 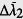 is, the larger 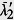 is; hence, the more synchronizable the entire network is. Therefore, we can add such an interlink that maximizes 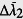, that is,


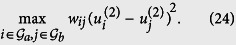


**Figure 7:**
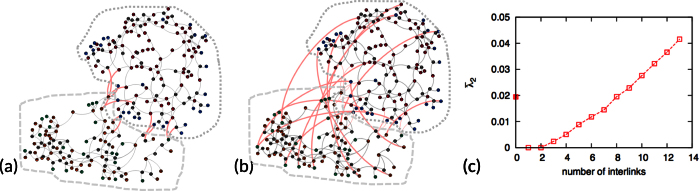
(**a**) The original Japan power grid network; (**b**) The optimized interlinks that connect the Tohoku and the Tokyo areas; (**c**) Comparison of 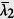 of the Laplacian matrix in the original (the filled square) and the optimized (the empty squares) networks. There are totally 13 interlinks connecting the Tohoku and the Tokyo areas.

In the Japan power grid network, there are totally 13 interlinks connecting the Tohoku area with the Tokyo area (see Supplementary Information S7). In order to evaluate whether the synchronization stability can be further improved by designing appropriate interlinks, we implemented an optimization algorithm (see Supplementary Information S6 for the details), and calculated the second-nonzero eigenvalue of the Laplacian matrix of the entire network, 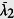. If we take the geographical distance between generators into account, after calculation, we found that almost all the present interlinks in the original network are optimally interconnected, which implies that the geographical distance is a fundamental factor when designing the real system. Therefore, we omit the result in the main text. Then, we turn to the unweighted case where *w*_*ij*_ = 1 for all the interlinks. In this case, the added interlinks are different from the original ones, see Fig. 7(a,b).

Spatial distance between machines seems to be a priority to be considered for the design of a real system (Fig. 7(a)), while the optimized interconnected network is spatially separated. The observation of spatial connections here is consistent with the global communication network as shown in Fig. 6. The optimized interconnected network takes an advantage at the improvement of the synchronizability. We observe that the improved 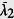 is approximately the same as that in the original network when adding only 8 links. By adding more interlinks, 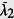 can be further improved up to twice as high as the original value (Fig. 7(c)).

**Figure 8:**
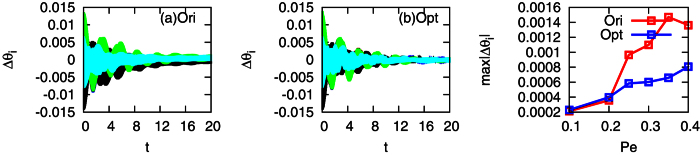
Comparison of the synchronization stability between the original (ori) and the optimized (opt) networks. (**a**) The difference of phases before and after perturbation in the original network; (**b**) The difference of phases before and after perturbation in the optimized network; (**c**) The maximum difference of the phases before and after perturbation in the original (the red squares) and the optimized networks (the blue squares), respectively, versus power demand *P*_*e*_, where *P*_*e*,*i*_ = *P*_*e*_ = 0.1 (red), 0.2 (blue), 0.3 (black), 0.35 (green), and 0.4 (cyan) for 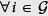. The perturbation was applied to the phase of each generator in the synchronous state at *t* = 0, and was drawn from the Gaussian distribution with mean zero and standard deviation 0.05 rad. Each point shows the averaged results of 200 times of random perturbations. The difference of the phases is defined as Δ*θ*_*i*_ = *θ*_*i*1_ − θ_*,*i*1_ for 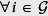, where θ_*,*i*_ is the synchronous state. The parameters are set at *H*_*a*,*i*_ = *H*_*b*,*i*_ = 2.5 for 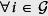.

In order to compare the synchronization stability between the optimized interconnected networks and the original network, we set all the parameters, such as the power demand *P*_*e*,*i*_, the inertia, and the damping coefficients, to be the same in the two networks, although the steady states of the two systems are different. Then, each phase of generator *i* at the synchronous state θ_*,*i*_ is perturbed at *t* = 0 following the Gaussian distribution with mean zero and standard deviation 0.05. To see whether the state can eventually return to the synchronized state, we measure the difference between the perturbed phase *θ*_*i*_ and the original synchronized phase θ_*,*i*_ in the two networks, respectively, denoted as Δ*θ*_*i*_ = *θ*_*i*1_ − θ_*,*i*1_, where *θ*_1_ is taken as a reference phase. Each synchronous state is obtained by using different power demands *P*_*e*,*i*_ = *P*_*e*_ = 0.1, 0.2, 0.3, 0.35, and 0.4. In Fig. 8(a,b), we observe that the optimized interconnected network maintains better synchronization stability, where all phases can return to their synchronous state with less difference Δ*θ*_*i*_ than that in the original network. To quantify the difference of the phase, we measure the maximum absolute value of Δ*θ*_*i*_, max |Δ*θ*_*i*_|, at the final stable state for all the power demand *P*_*e*_ we tested in Fig. 8(c), which shows that when the power demand *P*_*e*_ is small, max |Δ*θ*_*i*_| in the two networks are close, while with the increase of *P*_*e*_, max |Δ*θ*_*i*_| of the original network is larger than that of the optimized network, which indicates that the optimized network possesses better synchronization stability than the original network.

## Discussion

Network topology can play a key role in the network synchronization. Based on the observation that a power grid is often interconnected, we have revealed and analyzed the synchronization stability of coupled phase oscillators in an interconnected power grid network with the incorporation of different control strategies and the design of interlinks. For the self-feedback control strategy, the optimal control strength can be obtained by relating the system parameters such as the inertia coefficient, with the network structure at the steady state. For the local feedback control strategy, the optimal local communication network on subnetwork ‘a’, and that on subnetwork ‘b’, are built, respectively. Then, the global communication network that connects generators in subnetwork ‘a’ with those in subnetwork ‘b’ is built. We found that the global communication network can improve the synchronization stability most.

Relating the network interlinks with the synchronization state, we improved the network synchronization by changing the network interlinks. By testing the synchronization stability in the optimized network and the original network, respectively, for a number of synchronous states, we found that at lower power demands, the optimized network and the original network show similar stability; while at high power demands, the optimized network shows better synchronization stability than the original network does. This result highlights the role of the network interlinks in the synchronization of coupled oscillators. Both the optimized interconnected network and the optimized communication network show similar connectivity patterns, that is, connecting nodes that are spatially distant. In the present situation, the design of such an ideally stable power grid network seems unfeasible due to the enormous cost for building the long electric power lines. Therefore, while the network interlinks are optimal in the context of complex network theory, they may be hard to be implemented in practice. The design of real power grids seems to depend more on the spatial distance; the shorter geographical distance is preferred in the design of a power grid. In the near future, a model that balances the trade-off between spatial distance and synchronization stability would be expected.

## Additional Information

**How to cite this article**: Wang, B. *et al.* Enhancing synchronization stability in a multi-area power grid. *Sci. Rep.* **6**, 26596; doi: 10.1038/srep26596 (2016).

## Supplementary Material

Supplementary Information
