# Supplementary material for: Enhancing synchronization stability in a multi-area power grid
Source: Sci Rep. 2016 May 26;6:26596. doi: 10.1038/srep26596 (PMC4881022; doi:10.1038/srep26596)
Supplement: Supplementary Information [file srep26596-s1.pdf]

Supplementary Information for "Enhancing synchronization stability in a multi-area power grid"

Bing Wang,[1,2,*] Hideyuki Suzuki,[3] and Kazuyuki Aihara[2]

[1]*School of Computer Engineering and Science, Shanghai University, No.99 Shangda Road, Baoshan District, Shanghai 200444, P. R. China*

[2]*Institute of Industrial Science, The University of Tokyo, 4-6-1 Komaba, Meguro-ku, Tokyo 153-8505*

[3]*Graduate School of Information Science and Technology, The University of Tokyo, 7-3-1 Hongo, Bunkyo-ku, Tokyo 113-8656, Japan*

(Dated: April 14, 2016)

* bingbignmath@gmail.com, bingbignwang@shu.edu.cn

S.1. NETWORK REDUCTION

When analyzing the synchronization stability, the network has to be reduced to be composed only of generators; load nodes and branching points have to be removed from the network. This process is often named as Kron reduction [1]. According to Kirchhoff's law, we have $I = YV$, where I and V are the vectors of currents and voltages; Y is the admittance matrix. Since the non-generator nodes are regarded as constant impedances, their injection currents are zeros. By rearranging Y such that the first n indices correspond to the generator nodes and the remaining r corresponding to the non-generator nodes, we have

$$\begin{bmatrix} I_n \\ 0 \end{bmatrix} = \begin{bmatrix} Y_{n\times n} & Y_{n\times r} \\ Y_{r\times n} & Y_{r\times r} \end{bmatrix} \begin{bmatrix} V_n \\ V_r \end{bmatrix}. \tag{S1}$$

By removing V_r, the effective admittance matrix is given as

$$Y' = Y_{n\times n} - Y_{n\times r} Y_{r\times r}^{-1} Y_{r\times n}. \tag{S2}$$

S.2. THE POWER FLOW EQUATIONS

The swing equations for the entire system composed of two subnetworks 'a' and 'b' are given by

$$\begin{aligned}
\dot{\theta}_i^a &= \omega_i^a, \\
\dot{\omega}_i^a &= -\frac{D_{a,i}}{H_{a,i}}\omega_i^a + \frac{1}{H_{a,i}}\{P_{m,i}^a - |V_i^a| \sum_{k\in\mathcal{N}_a\backslash\{i\}} |V_k^a|(G_{ik}^{aa}\cos\theta_{ik}^{aa} + B_{ik}^{aa}\sin\theta_{ik}^{aa}) \\
&\quad -|V_i^a| \sum_{k\in\mathcal{N}_b} |V_k^b|(G_{ik}^{ab}\cos\theta_{ik}^{ab} + B_{ik}^{ab}\sin\theta_{ik}^{ab})\}, \\
\dot{\theta}_j^b &= \omega_j^b, \\
\dot{\omega}_j^b &= -\frac{D_{b,j}}{H_{b,j}}\omega_j^a + \frac{1}{H_{b,j}}\{P_{m,j}^b - |V_j^b| \sum_{k\in\mathcal{N}_b\backslash\{j\}} |V_k^b|(G_{jk}^{bb}\cos\theta_{jk}^{bb} + B_{jk}^{bb}\sin\theta_{jk}^{bb}) \\
&\quad -|V_j^b| \sum_{k\in\mathcal{N}_a} |V_k^a|(G_{jk}^{ba}\cos\theta_{jk}^{ba} + B_{jk}^{ba}\sin\theta_{jk}^{ba})\},
\end{aligned} \tag{S3}$$

for $i \in \mathcal{G}_a$ and $j \in \mathcal{G}_b$.

Since generators are controlled by governors, we have to consider the dynamics of generators and loads separately. The consumed electric power at load i in subnetwork 'a' is represented by $P_{e,i}^a$ and $Q_{e,i}^a$, which are the active and reactive power, respectively. For each

load $i \in \mathcal{L}$, where $\mathcal{L}$ denotes the set of loads in the network, the power-balanced condition should be satisfied as follows:

$$
\begin{aligned}
P_{e,i}^{a} &= |V_i^a|\{\sum_{k\in\mathcal{N}_a\backslash\{i\}} |V_k^a|(G_{ik}^{aa}\cos\theta_{ik}^{aa} + B_{ik}^{aa}\sin\theta_{ik}^{aa}) + \sum_{k\in\mathcal{N}_b} |V_k^b|(G_{ik}^{ab}\cos\theta_{ik}^{ab} + B_{ik}^{ab}\sin\theta_{ik}^{ab})\},\\
Q_{e,i}^{a} &= |V_i^a|\{\sum_{k\in\mathcal{N}_a\backslash\{i\}} (|V_k^a|(G_{ik}^{aa}\sin\theta_{ik}^{aa} - B_{ik}^{aa}\cos\theta_{ik}^{aa}) + \sum_{k\in\mathcal{N}_b} |V_k^b|(G_{ik}^{ab}\sin\theta_{ik}^{aa} - B_{ik}^{ab}\cos\theta_{ik}^{ab})\},\\
P_{e,j}^{b} &= |V_j^b|\{\sum_{k\in\mathcal{N}_b\backslash\{j\}} |V_k^b|(G_{jk}^{bb}\cos\theta_{jk}^{bb} + B_{jk}^{bb}\sin\theta_{jk}^{bb}) + \sum_{k\in\mathcal{N}_a} |V_k^a|(G_{jk}^{ba}\cos\theta_{jk}^{ba} + B_{jk}^{ba}\sin\theta_{jk}^{ba})\},\\
Q_{e,j}^{b} &= |V_j^b|\{\sum_{k\in\mathcal{N}_b\backslash\{j\}} |V_k^b|(G_{jk}^{bb}\sin\theta_{jk}^{bb} - B_{jk}^{bb}\cos\theta_{jk}^{bb}) + \sum_{k\in\mathcal{N}_a} |V_k^a|(G_{jk}^{ba}\sin\theta_{jk}^{ba} - B_{jk}^{ba}\cos\theta_{jk}^{ba})\},
\end{aligned}
\tag{S4}
$$

for $i \in \mathcal{L}_a$ and $j \in \mathcal{L}_b$. The sets of nodes in subnetwork 'a' and subnetwork 'b' are denoted by $\mathcal{N}_a = \mathcal{G}_a \cup \mathcal{L}_a$ and $\mathcal{N}_b = \mathcal{G}_b \cup \mathcal{L}_b$, respectively.

Equation (S4) contains $2n_a+2n_b$ variables. Given the admittance matrix $Y_{ik} = G_{ik}+jB_{ik}$ with $j^2 = -1$ and the parameters $P_{e,i}^a$, $Q_{e,i}^a$, $P_{e,i}^b$, and $Q_{e,i}^b$ for $i \in \mathcal{L}$, the voltage $|V_i|$ and the phase θ_i, for $\forall i \in \mathcal{L}$, can be calculated by the Newton-Raphson method, where the voltages and the phases of the generators are calculated with equation (S3) by the Runge-Kutta method. The voltages of generators are assumed to be constant since constant voltage magnitude is usually maintained by voltage regulators.

S.3. THE EQUILIBRIUM SOLUTION OF THE SWING EQUATIONS FOR THE TWO INTERCONNECTED SUBNETWORKS

We denote the equilibrium solution of equation (S3) as $(\theta_{*,i}^a, \omega_{*,i}^a, \theta_{*,j}^b, \omega_{*,j}^b)$ for $i \in \mathcal{G}_a$ and $j \in \mathcal{G}_b$, and $(\theta_i^a, \omega_i^a, \theta_j^b, \omega_j^b)$ is the state obtained by the perturbation around the equilibrium as follows:

$$
\begin{aligned}
\theta_i^a &= \theta_{*,i}^a + \Delta\theta_i^a,\\
\omega_i^a &= \omega_{*,i}^a + \Delta\omega_i^a,\\
\theta_j^b &= \theta_{*,j}^b + \Delta\theta_j^b,\\
\omega_j^b &= \omega_{*,j}^b + \Delta\omega_j^b.
\end{aligned}
$$

For simplicity, we assume that the transmission line is lossless, i.e., $G_{ik}^{aa} = G_{ik}^{bb} = G_{ik}^{ab} = G_{ik}^{ba} = 0$. The linearized equation around the equilibrium is given by

$$\begin{aligned}
&\dot{\Delta\theta}_i^a = \Delta\omega_i^a, \\
&\dot{\Delta\omega}_i^a = -\frac{D_{a,i}}{H_{a,i}}\Delta\omega_i^a - \frac{1}{H_{a,i}}(\gamma_a\Delta\theta_i^a + \sum_{k\in\mathcal{G}_a\backslash\{i\}} B_{ik}^{aa}\cos\theta_{*,ik}^{aa}\Delta\theta_{ik}^{aa} + \sum_{k\in\mathcal{G}_b} B_{ik}^{ab}\cos\theta_{*,ik}^{ab}\Delta\theta_{ik}^{ab}). \quad \text{(S5)}
\end{aligned}$$

The differential equations for generators j in subnetwork 'b' can be expressed in a similar way as follows:

$$\begin{aligned}
&\dot{\Delta\theta}_j^b = \Delta\omega_j^b, \\
&\dot{\Delta\omega}_j^b = -\frac{D_{b,j}}{H_{b,j}}\Delta\omega_j^b - \frac{1}{H_{b,j}}(\gamma_b\Delta\theta_j^b + \sum_{k\in\mathcal{G}_b\backslash\{j\}} B_{jk}^{bb}\cos\theta_{*,jk}^{bb}\Delta\theta_{jk}^{bb} + \sum_{k\in\mathcal{G}_a} B_{jk}^{ba}\cos\theta_{*,jk}^{ba}\Delta\theta_{jk}^{ba}), \quad \text{(S6)}
\end{aligned}$$

for $\forall j \in \mathcal{G}_b$.

S.4. THE STEADY-STATE STABILITY WITH SELF-FEEDBACK CONTROL

The self-feedback control matrix $\mathbf{K}$ and the damping matrix $\mathbf{M}$ are defined as

$$\mathbf{K} = \begin{bmatrix} \mathbf{K^a} & \mathbf{0} \\ \mathbf{0} & \mathbf{K^b} \end{bmatrix} = \begin{bmatrix} \frac{\gamma_{a,i}}{H_{a,i}}\mathbf{I} & 0 \\ 0 & \frac{\gamma_{b,j}}{H_{b,j}}\mathbf{I} \end{bmatrix}, \quad \text{for } i \in \mathcal{G}_a, j \in \mathcal{G}_b, \quad \text{(S7)}$$

$$\mathbf{M} = \begin{bmatrix} \mathbf{M^a} & \mathbf{0} \\ \mathbf{0} & \mathbf{M^b} \end{bmatrix} = \begin{bmatrix} \frac{D_{a,i}}{H_{a,i}}\mathbf{I} & 0 \\ 0 & \frac{D_{b,j}}{H_{b,j}}\mathbf{I} \end{bmatrix}, \quad \text{for } i \in \mathcal{G}_a, j \in \mathcal{G}_b. \quad \text{(S8)}$$

The eigenvalue of the matrix $\mathbf{K}$, $\lambda_{K,i}$ is given as follows:

$$\lambda_{K,i} = \begin{cases} \frac{\gamma_a}{H_{a,i}} & \forall i \in \mathcal{G}_a, \\ \frac{\gamma_b}{H_{b,i}}, & \forall i \in \mathcal{G}_b, \end{cases}$$

where the control strength for each subarea is assumed to be the same as $\gamma_{a,i} = \gamma_a$ and $\gamma_{b,i} = \gamma_b$, respectively. In order to conduct the analysis, we further assume that $\lambda_{K,i}$ can be adjusted to a common value as λ_K. By changing the scale of variables, the elements of the matrices $\mathbf{M^a}$ and $\mathbf{M^b}$ can also be adjusted to a common value $\lambda_M = \frac{\bar{D}}{H}$.

Denote the matrix $\mathbf{L}$ as

$$\mathbf{L} = \begin{bmatrix} 0 & 1 \\ (-\lambda_{C,i} + \lambda_K) & -\lambda_M \end{bmatrix}, \text{ for } i = 1, \ldots, n_a + n_b. \quad \text{(S9)}$$

The synchronization stability is then determined by the following eigenvalues :

$$\lambda_{\pm,i} = \frac{-\lambda_M \pm \sqrt{\lambda_M^2 - 4(\lambda_{C,i} - \lambda_K)}}{2}, \quad \text{for } i = 1, \ldots, n_a + n_b. \quad \text{(S10)}$$

S.5. THE STEADY-STATE ANALYSIS WITH THE LOCAL- AND GLOBAL-FEEDBACK CONTROL IN COMMUNICATION NETWORKS

The communication matrix of the local feedback control in subnetwork 'a', $\tilde{A}^{aa}_{n_a \times n_a}$, is defined as:

$$\tilde{A}^{aa}_{ik} = \begin{cases} 1 & i \triangleright k, \\ 0 & \text{otherwise}, \end{cases}$$

where $i \triangleright k$ indicates that generator i is connected with generator k by the communication network of subnetwork 'a'. The mechanical power input to generator i in subnetwork 'a' is then given by

$$\frac{dP^a_{m,i}}{dt} = -\gamma_a \sum_{k \in \mathcal{G}_a} \tilde{A}^{aa}_{ik} \frac{d\theta^{aa}_{ik}}{dt}, \forall i \in \mathcal{G}_a. \tag{S11}$$

Our goal is to find a matrix $\tilde{A}^{aa}$ such that the synchronization stability can be improved most.

The linearized swing equation for generator i in subnetwork 'a' is given by

$$\begin{aligned} &\Delta\dot{\theta}^a_i = \Delta\omega^a_i, \\ &\Delta\dot{\omega}^a_i = -\frac{D_{a,i}}{H_{a,i}}\Delta\omega^a_i - \frac{1}{H_{a,i}}(\gamma_a \sum_{k \in \mathcal{G}_a \backslash \{i\}} \tilde{A}^{aa}_{ik}\Delta\theta^{aa}_{ik} + \sum_{k \in \mathcal{G}_a} B^{aa}_{ik}\cos\theta^{aa}_{*,ik}\Delta\theta^{aa}_{ik} + \sum_{k \in \mathcal{G}_b} B^{ab}_{ik}\cos\theta^{ab}_{*,ik}\Delta\theta^{ab}_{ik}), \end{aligned} \tag{S12}$$

for $\forall i \in \mathcal{G}_a$.

By doing similar analysis as we did in the self-feedback control, we obtain an equation for variables $\mathbf{X_1}$ and $\mathbf{X_2}$ as follows:

$$\begin{bmatrix} \dot{\mathbf{X}}_1 \\ \dot{\mathbf{X}}_2 \end{bmatrix} = \begin{bmatrix} \mathbf{0} & \mathbf{I} \\ -\mathbf{C} - \mathbf{K} & -\mathbf{M} \end{bmatrix} \begin{bmatrix} \mathbf{X_1} \\ \mathbf{X_2} \end{bmatrix}, \tag{S13}$$

where the form of the control matrix $\mathbf{K}$ can be expressed as:

$$\mathbf{K} = \begin{bmatrix} \mathbf{K^{aa}} & \mathbf{0} \\ \mathbf{0} & \mathbf{0} \end{bmatrix}. \tag{S14}$$

By diagonalizing the matrix $\mathbf{C} + \mathbf{K}$, we obtain $\mathbf{J_{CK}} = \mathbf{Q^{-1}_{CK}}(\mathbf{C} + \mathbf{K})\mathbf{Q_{CK}}$, where $\mathbf{Q_{CK}}$ is composed of the eigenvectors. The eigenvalues of $\mathbf{J_{CK}}$ are ordered as $0 = \lambda_{CK,1} \leq \cdots \leq \lambda_{CK,n_a+n_b}$. By setting $\mathbf{Z_1} = \mathbf{Q^{-1}_{CK}}\mathbf{X_1}$ and $\mathbf{Z_2} = \mathbf{Q^{-1}_{CK}}\mathbf{X_2}$, respectively, we have

$$\begin{bmatrix} \dot{\mathbf{Z}}_1 \\ \dot{\mathbf{Z}}_2 \end{bmatrix} = \begin{bmatrix} \mathbf{0} & \mathbf{I} \\ -\mathbf{J_{CK}} & -\mathbf{M} \end{bmatrix} \begin{bmatrix} \mathbf{Z_1} \\ \mathbf{Z_2} \end{bmatrix}. \tag{S15}$$

We denote the matrix $\mathbf{L}$ as

$$\mathbf{L} = \begin{bmatrix} \mathbf{0} & \mathbf{I} \\ -\mathbf{J_{CK}} & -\mathbf{M} \end{bmatrix}. \tag{S16}$$

Under the interaction of the network topology and the communication structure, the synchronization stability is determined by the eigenvalues of $\mathbf{L}$,

$$\lambda_{\pm,i} = \frac{-\lambda_M \pm \sqrt{\lambda_M^2 - 4\lambda_{CK,i}}}{2}, \quad \text{for} \quad i = 2, \ldots, n_a + n_b. \tag{S17}$$

The expression of the matrix $\mathbf{K}$ relies on different local strategies. For instance, if a communication network is built in subnetwork 'b', denoted by $\tilde{A}^{bb}$, the local feedback control matrix $\mathbf{K}$ is given by

$$\mathbf{K} = \begin{bmatrix} \mathbf{0} & \mathbf{0} \\ \mathbf{0} & \mathbf{K^{bb}} \end{bmatrix}, \tag{S18}$$

where $\mathbf{K^{bb}}$ is defined in a similar way as $\mathbf{K^{aa}}$, given by

$$\mathbf{K^{bb}}_{ik} = \begin{cases} \frac{\gamma_b}{H_{b,i}} \tilde{A}^{bb}_{ik} & k \neq i, \\ -\sum_{k' \in \mathcal{G}_b \setminus \{i\}} \mathbf{K^{bb}}_{ik'}, & \text{otherwise}, \end{cases} \tag{S19}$$

for $i, k \in \mathcal{G}_b$.

The general expression of the matrix $\mathbf{K}$ can be expressed as:

$$\mathbf{K} = \begin{bmatrix} \mathbf{K^{aa}} & \mathbf{K^{ab}} \\ \mathbf{K^{ba}} & \mathbf{K^{bb}} \end{bmatrix}. \tag{S20}$$

If a global control of generators is implemented, a global communication matrix that connects generators in subnetwork 'a' with generators in subnetwork 'b' is established. Then, the matrix $\tilde{A}^{ab}_{n_a \times n_b}$ is defined as:

$$\tilde{A}^{ab}_{ik} = \begin{cases} 1 & i \triangleright k, \\ 0 & \text{otherwise}, \end{cases}$$

for $i \in \mathcal{G}_a, k \in \mathcal{G}_b$. The matrix $\mathbf{K^{ab}}$ is an $n_a \times n_b$ matrix with $\mathbf{K^{ab}}_{ik} = \frac{\gamma_{ab}}{H_{a,i}} \tilde{A}^{ab}_{ik}$ for $i \in \mathcal{G}_a, k \in \mathcal{G}_b$, where γ_{ab} represents the control strength of generators locating in subnetworks 'a' and 'b'. The matrix $\mathbf{K^{aa}}_{n_a \times n_a}$ is a diagonal matrix composed of the element $\mathbf{K^{aa}}_{ii} = -\frac{\gamma_{ab}}{H_{a,i}} \sum_{k \in \mathcal{G}_b} \tilde{A}^{ab}_{ik}$ for $i \in \mathcal{G}_a$. The matrix $\tilde{A}^{ba}_{n_b \times n_a}$ and the matrix $\mathbf{K^{ba}}_{n_b \times n_a}$ can be defined in a similar way.

Here, we only present the algorithm for the optimal communication network in subnetwork 'a', $\tilde{A}^{aa}$. The algorithm can be applied to the optimal communication network

in subnetwork 'b' and the global communication network in a similar way. Assume that there are totally m communication links that will be built in subnetwork 'a', the process of establishing the communication network is described as follows:

1. Initially, start with the subnetwork 'a', which is only composed of generators. Set $\tilde{A}^{aa}$ as a zero matrix and calculate Λ_{max} of the matrix $\mathbf{L}$ (equation (16));
2. Calculate Λ'_{max} of the matrix $\mathbf{L}'$, where the matrix $\tilde{A'}^{aa}$ is established by connecting generators i and k in subnetwork 'a';
3. Choose the pair of generators i and j that minimizes Λ'_{max} and connect them. Update the matrix $\tilde{A'}^{aa}$;
4. Return to step 2 until m edges are added and stop.

S.6. ENHANCEMENT OF SYNCHRONIZATION STABILITY BY CHANGING THE INTERLINKS

In order to understand the impact of network interlinks on the synchronization stability, we investigate the improvement of the synchronization stability by changing the network interlinks. The Laplacian matrix $W = D - A$, where D is the diagonal matrix with the row sums of A as the diagonal elements, and A is the adjacy matrix of the network. Consider that W has a simple zero eigenvalue and the eigenvalues of W have nonnegative real parts. The ascending order of the real parts of the eigenvalues is given as $0 = \bar{\lambda}_1 < \bar{\lambda}_2 \leq \cdots \leq \bar{\lambda}_n$. The larger $\bar{\lambda}_2$ is, the more synchronously stable the network is.

In the following, we apply perturbation analysis to improve $\bar{\lambda}_2$ by adding interlinks appropriately in the two interconnected networks. Assume that the weight of a link connecting nodes i and j is $w_{ij} > 0$. When the two subnetworks 'a' and 'b' are isolated, the weighted Laplacian matrix is given by

$$W = \begin{bmatrix} W^{\mathbf{a}} & \mathbf{0} \\ \mathbf{0} & W^{\mathbf{b}} \end{bmatrix}, \tag{S21}$$

where $W^{\mathbf{a}}$ and $W^{\mathbf{b}}$ represent the weighted Laplacian matrices of the subnetworks 'a' and 'b', respectively. Since W is a real symmetric matrix, it has $n_a + n_b$ real eigenvalues, which are ordered as $0 = \bar{\lambda}_1 = \bar{\lambda}_2 \leq \bar{\lambda}_3 \leq \cdots \leq \bar{\lambda}_{n_a+n_b}$, where 0 is the eigenvalue with multiplication 2 due to the two isolated subnetworks with the eigenvectors all of whose components are 1.

When adding a new interlink between the two subnetworks, the new Laplacian matrix of the network W' is a perturbed matrix of W, expressed as

$$W' = W + \mathbf{\Delta}\, W = \begin{bmatrix} W^{\mathbf{a}} + \mathbf{D_\Psi} & -\mathbf{\Psi} \\ -\mathbf{\Psi} & W^{\mathbf{b}} + \mathbf{D_\Psi} \end{bmatrix}, \tag{S22}$$

where

$$\mathbf{\Delta}\, W = \begin{bmatrix} \mathbf{D_\Psi} & -\mathbf{\Psi} \\ -\mathbf{\Psi} & \mathbf{D_\Psi} \end{bmatrix}, \tag{S23}$$

represents the perturbed matrix; the matrix $\mathbf{\Psi}_{ij} = w_{ij}$ if nodes i and j are interconnected; the matrix $\mathbf{D_\Psi}$ is a diagonal matrix with element $(\mathbf{D_\Psi})_{ii} = \sum_j w_{ij}$. For instance, by adding a new interlink, the perturbed Laplacian matrix $\mathbf{\Delta}\, W$ is

$$\mathbf{\Delta}\, W = \begin{bmatrix} \mathbf{D_\Psi} & -\mathbf{\Psi} \\ -\mathbf{\Psi} & \mathbf{D_\Psi} \end{bmatrix} = \begin{bmatrix} w_{ij} & \dots & -w_{ij} & \dots \\ \vdots & \ddots & \vdots & \vdots \\ -w_{ij} & \dots & w_{ij} & \dots \\ \vdots & \dots & \vdots & \ddots \end{bmatrix}. \tag{S24}$$

The second nonzero eigenvalue of W' is perturbed around $\bar{\lambda}_2$, i.e., $\bar{\lambda}_2'(\epsilon) = \bar{\lambda}_2 + \Delta\bar{\lambda}_2\epsilon + O(\epsilon)$, where ϵ is the coupling strength. By setting the eigenvector of $\bar{\lambda}_2$ as $u^{(2)} = (u_1^{(2)}, \dots, u_{n_a}^{(2)}, \dots, u_{n_a+n_b}^{(2)})$, where the superscription denotes the Fiedler vector while the subscription denotes the node index. We have

$$\triangle\bar{\lambda}_2 = \frac{< u^{(2)}, \mathbf{\Delta}\, W u^{(2)} >}{< u^{(2)}, u^{(2)} >}, \tag{S25}$$

where $< . >$ is the Euclidean inner product. Since $\mathbf{\Delta}\, W$ is semi-definite, we have $\Delta\bar{\lambda}_2 \geq 0$. The larger $\Delta\bar{\lambda}_2$ is, the larger $\bar{\lambda}_2'$ is; hence, the more synchronizable the entire network is. Therefore, we can add such an interlink that maximizes $\Delta\bar{\lambda}_2$, that is,

$$\max_{i\in\mathcal{G}_a, j\in\mathcal{G}_b} w_{ij}(u_i^{(2)} - u_j^{(2)})^2. \tag{S26}$$

Initially, the two subnetworks are isolated, then 0 is the eigenvalue with multiplicity 2. However, if we fix $u^{(1)} = (1, 1, \dots, 1)$, then $u_i^{(2)} - u_j^{(2)}$ is constant for arbitrary $i \in \mathcal{G}_a$ and $j \in \mathcal{G}_b$. Therefore, we can choose the interlink with the largest value $w_{ij}(u_i^{(2)} - u_j^{(2)})^2$.

The algorithm for finding the optimal interlinks between two subnetwork is described as follows:

1. Start with two isolated subnetworks and calculate the eigenvalue $\bar{\lambda}_2$ of the Laplacian matrix W of the entire network.

2. Add one interlink that connect with node i and k that maximizes $w_{ik}(u_i^{(2)} - u_k^{(2)})^2$. Save the new Laplacian matrix as W'.

3. Calculate $\bar{\lambda}_2'$ of W'.

4. Return to step 2 until the number of interlinks is satisfied and stop.

S.7. THE EASTERN JAPAN POWER GRID

The power grid network [2] is composed of the Tokyo area and the Tohoku area. They are connected by 13 interlinks. Regarding the nodes, there are totally 248 nodes, among which there are 53 generators, 126 load nodes, and 69 branching points, respectively, in the network. In the numerical simulation, without explicit specification, the parameters are set as $D_i = 1$ for $\forall i \in \mathcal{G}$ and the conductance matrix $G = 0$. The voltages of all the generators are assumed to be 1. The susceptance matrix B is set as

$$B_{ik} = \begin{cases} 10 & \text{if } i \text{ connects with } k, \\ 0 & \text{otherwise.} \end{cases} \tag{S27}$$

[1] Döfler, F. & Bullo, F. Synchronization of power networks: network reduction and effective resistance. *Proc. of the 2nd IFAC Workshop on Distributed Estimation and Control in Networked Systems, Annecy, France* 197–202 (2010).

[2] Nagata, M. *et al.* Node-wise robustness against fluctuations of power transmission in power grids. *Eur. Phys. J. Special Topics* **223**, 2549–2559 (2014).
